# Recent advances in targeted therapies in acute myeloid leukemia

**DOI:** 10.1186/s13045-023-01424-6

**Published:** 2023-03-25

**Authors:** Rahul S. Bhansali, Keith W. Pratz, Catherine Lai

**Affiliations:** Division of Hematology/Oncology, Department of Medicine, Hospital of the University of Pennsylvania, South Pavilion, 12th Floor, 3400 Civic Center Blvd, Philadelphia, PA 19104 USA

**Keywords:** Acute myeloid leukemia, Targeted therapy, Novel treatments, Combination therapy, FLT3, IDH1, IDH2, TP53

## Abstract

Acute myeloid leukemia (AML) is the most common acute leukemia in adults. While survival for younger patients over the last several decades has improved nearly sixfold with the optimization of intensive induction chemotherapy and allogeneic stem cell transplantation (alloHSCT), this effect has been largely mitigated in older and less fit patients as well as those with adverse-risk disease characteristics. However, the last 10 years has been marked by major advances in the molecular profiling of AML characterized by a deeper understanding of disease pathobiology and therapeutic vulnerabilities. In this regard, the classification of AML subtypes has recently evolved from a morphologic to a molecular and genetic basis, reflected by recent updates from the World Health Organization and the new International Consensus Classification system. After years of stagnation in new drug approvals for AML, there has been a rapid expansion of the armamentarium against this disease since 2017. Low-intensity induction therapy with hypomethylating agents and venetoclax has substantially improved outcomes, including in those previously considered to have a poor prognosis. Furthermore, targeted oral therapies against driver mutations in AML have been added to the repertoire. But with an accelerated increase in treatment options, several questions arise such as how to best sequence therapy, how to combine therapies, and if there is a role for maintenance therapy in those who achieve remission and cannot undergo alloHSCT. Moreover, certain subtypes of AML, such as those with *TP53* mutations, still have dismal outcomes despite these recent advances, underscoring an ongoing unmet need and opportunity for translational advances. In this review, we will discuss recent updates in the classification and risk stratification of AML, explore the literature regarding low-intensity and novel oral combination therapies, and briefly highlight investigative agents currently in early clinical development for high-risk disease subtypes.

## Background

Acute myeloid leukemia (AML) is the most common acute leukemia in adults. AML is thought to arise from somatically acquired mutations, which is a fairly ubiquitous process during human aging [1–11]. However, AML can arise both de novo and secondary to other processes including antecedent hematologic disorders or exposure to immunosuppressive or cytotoxic therapies. The median age of diagnosis of AML is 68 years in the USA, and the incidence continues to increase with age [12]. The median overall survival (OS) from diagnosis for patients under the age of 65 had improved from 8 months between 1975 and 1979 to 46 months between 2010 and 2014; however, survival in patients older than 65 has only marginally improved during this same interval [12, 13]. This is in part because induction with intensive cytarabine- and anthracycline-based chemotherapy (i.e., “7 + 3”) has remained the standard of care for AML for over 40 years, the tolerability of which is limited in older and less fit patients.

**Fig. 1:**
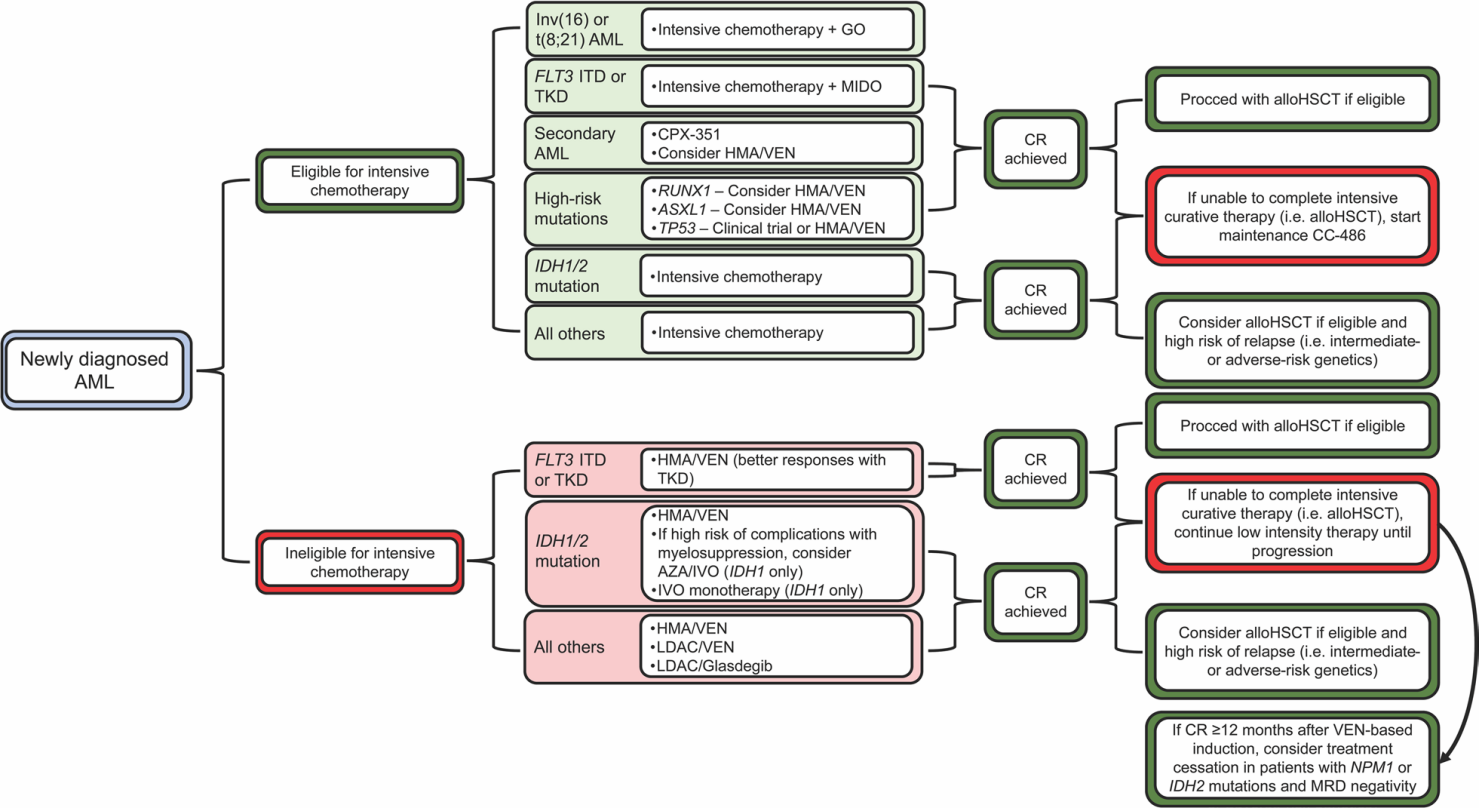
Approach to frontline treatment of AML with FDA-approved therapies in 2022. Treatment algorithm of AML induction and maintenance therapy is shown. *AML* Acute myeloid leukemia, *GO* Gemtuzumab ozogamicin, *MIDO* Midostaurin, *HMA* Hypomethylating agent, *VEN* Venetoclax, *ENA* Enasidenib; *IVO* Ivosidenib, *LDAC* Low-dose cytarabine, *alloHSCT* Allogeneic stem cell transplantation, *MRD* Measurable residual disease, *CR* Complete remission

While optimization of intensive chemotherapy and better supportive care over the years has improved survival in AML, this benefit is largely confined to younger patients and those without adverse-risk cytogenetics. Moreover, the only potentially curative strategy for those with intermediate- or adverse-risk disease is allogeneic hematopoietic stem cell transplantation (alloHSCT) [14], which is not an option for many patients with AML due to age, frailty, and medical co-morbidities [15]. Accordingly, there has been a considerable interest in de-intensifying induction therapy, guided by an improved understanding of AML pathobiology due to advances in genomic profiling [16–18]. This has led to the approval of multiple novel agents and targeted therapies, which are now increasingly employed in the frontline, relapsed/refractory (R/R), and maintenance settings (reviewed in [19–22]). However, with 10 new Food and Drug Administration (FDA) approvals for AML in the last 5 years and increasing availability of personalized genomic data, important questions arise of how to best personalize the treatment of patients with AML and how to utilize transplantation in the context of targeted therapies (Fig. 1). In this review, we will discuss the recent updates in classification and risk stratification of AML, explore combination therapies in clinical practice and situations in which intensive chemotherapy can potentially be replaced, and highlight ongoing areas of investigation for AML therapeutics.

## Updates in classification and risk stratification of AML

**Fig. 2:**
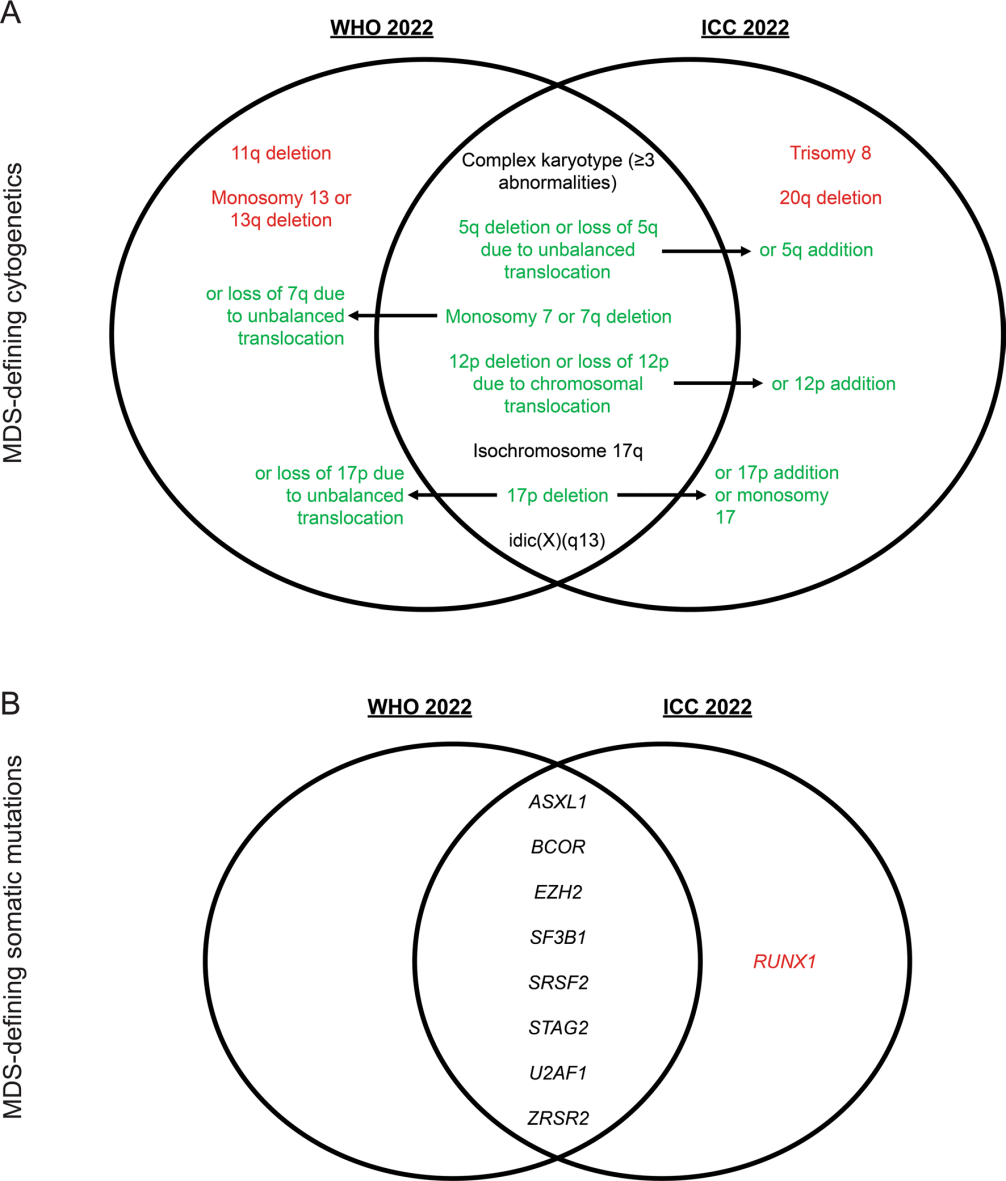
Updates in WHO/ICC MDS-defining genetic alterations in AML. Venn diagrams depict overlapping and distinct MDS-defining cytogenetic alterations **A** and somatic mutations **B** determined by the WHO [23] and ICC [24] Green text with arrows denotes overlapping entities with minor differences, black text denotes completely overlapping entities, and red text denotes completely distinct entities. *MDS* Myelodysplastic syndrome *WHO* World Health Organization, *ICC* International Consensus Classification

In 2022, the World Health Organization (WHO) [23] updated their classification of hematolymphoid neoplasms, and a separate International Consensus Classification (ICC) system was formed [24]. While these have led to changes in many hematologic malignancies, we will henceforth focus on changes related to AML (summarized in Table 1). Many disease entities remain the same, and most changes revolve around incorporating cytogenetic and genetic information into diagnostic classification. A significant change is that myelodysplastic syndrome with excess blasts 2 (MDS-EB2) is no longer a recognized MDS subtype, reflecting a spectrum of disease between MDS and AML rather than a discrete transition at the arbitrary cutoff of 20% blasts. The WHO allows for a diagnosis of AML with a blast count below 20% if there are defining genetic abnormalities with the exception of *BCR::ABL1* fusions and *CEBPA* mutations. Cases of AML without a defining genetic alteration are made based on differentiation patterns and still require a blast count of at least 20%. Similarly, the ICC uses a 10% blast cutoff for most molecularly defined subtypes of AML, again with the exception of *BCR::ABL1* to avoid confusion with the diagnosis of chronic myeloid leukemia (CML)*.* Moreover, both the WHO and ICC define cytogenetic and genetic changes almost always associated with antecedent MDS (Fig. 2A–B), while the cytogenetic profile has not drastically changed with a few exceptions, mutations in *ASXL1*, *BCOR*, *EZH2, SF3B1, SRSF2*, *STAG2*, *U2AF1*, or *ZRSR2* now define AML with myelodysplasia-related gene mutations given the high prevalence of these mutations in MDS; *RUNX1* mutations are also considered MDS-defining by the ICC but not by the WHO [25–28]. By relying on molecular rather than morphologic classification of AML, this may allow for nuanced prognostication and definition of targets for measurable residual disease (MRD) monitoring during treatment, though there is still no standard of how to incorporate this into routine care. Two major differences between the updated WHO and ICC systems are how AML with *CEBPA* and *TP53* alterations are defined. Several studies have now shown that basic leucine zipper (bZIP) domain mutations in *CEBPA* confer a better prognosis with a distinct gene expression profile [29–31], so this is now an AML-defining genetic alteration in both classification systems, though the WHO still also includes other bi-allelic *CEBPA* mutations, while the ICC does not. Furthermore, the ICC defines *TP53*-mutated AML and MDS/AML as a distinct genetic entity due to the characteristically poor prognosis associated with this mutation [32–34]. Any somatic *TP53* mutation with a variant allelic frequency (VAF) above 10% now defines this subtype of MDS/AML or AML. The WHO system created a distinct entity for *TP53*-mutated AML due to the frequent co-occurrence with complex cytogenetics or therapy-related AML (tAML).

**Table 1:** Updates in WHO/ICC classifications of AML

	WHO 2022	ICC 2022*
AML with defining genetic abnormalities**	APL with *PML::RARA* fusion	APL with t (15;17) (q24.1;q21.2)/*PML::RARA*^§^
		APL with other *RARA* rearrangements^§^
	AML with *RUNX1::RUNX1T1* fusion	AML with t (8;21) (q22;q22.1)/*RUNX1::RUNX1T1*^§^
	AML with *CBFB::MYH11* fusion	AML with inv (16) (p13.1q22) or t (16;16) (p13.1;q22)/*CBFB::MYH11*^§^
	AML with *DEK::NUP214* fusion	AML with t (6;9) (p22.3;q34.1)/*DEK::NUP214*^§^
	AML with *RBM15::MRTFA* fusion	*Not recognized*
	AML with *BCR::ABL1* fusion	AML with t (9;22) (q34.1;q11.2)/*BCR::ABL1*^#^
	AML with *KMT2A* rearrangement	AML with t (9;11) (p21.3;q23.3)/*MLLT3::KMT2A*^§^
		AML with other *KMT2A* rearrangements^§^
	AML with *MECOM* rearrangement	AML with inv (3) (q21.3q26.2) or t (3;3) (q21.3;q26.2)/*GATA2; MECOM (EVI1)*^§^
		AML with other *MECOM* rearrangements^§^
	AML with *NUP98* rearrangement	*Not recognized*
	AML with *NPM1* mutation	AML with mutated *NPM1*^§^
	AML with *CEBPA* mutation	AML with in-frame bZIP *CEBPA* mutations^§^
	AML, myelodysplasia-related^†^	AML^#^ and MDS/AML^§^ with mutated *TP53*
		AML^#^ and MDS/AML^§^ with myelodysplasia-related gene mutations
		AML^#^ and MDS/AML^§^ with myelodysplasia-related cytogenetic abnormalities
		MDS/AML NOS^§^
	AML with other defined genetic alterations	AML with other rare recurring translocations^#^
		Myeloid proliferations associated with Down syndrome
AML, defined by differentiation	AML with minimal differentiation	AML NOS^#^
	AML without maturation	
	AML with maturation	
	Acute basophilic leukemia	
	Acute myelomonocytic leukemia	
	Acute monocytic leukemia	
	Acute erythroid leukemia	
	Acute megakaryoblastic leukemia	
	Myeloid sarcoma	Myeloid sarcoma
	Blastic plasmacytoid dendritic cell neoplasm	Blastic plasmacytoid dendritic cell neoplasm

*AML* Acute myeloid leukemia, *APL* Acute promyelocytic leukemia, *MDS* Myelodysplastic syndrome, *bZIP* Basic leucine zipper domain, *NOS* Not otherwise specified, *WHO* World Health Organization, *ICC* International Consensus Classification

^*^Requires mention of qualifiers (Therapy-related, Progressing from MDS, Progressing from MDS/MPN, and/or Germline predisposition)

^**^ ≥ 20% blast cutoff is no longer required for AML with defining genetic abnormalities except for *BCR::ABL* fusion and *CEBPA* mutation

^†^ AML, myelodysplasia-related encompasses AML transformation from MDS and MDS/MPN

^§^Blast cutoff ≥ 10%

^#^Blast cutoff ≥ 20%

**Fig. 3:**
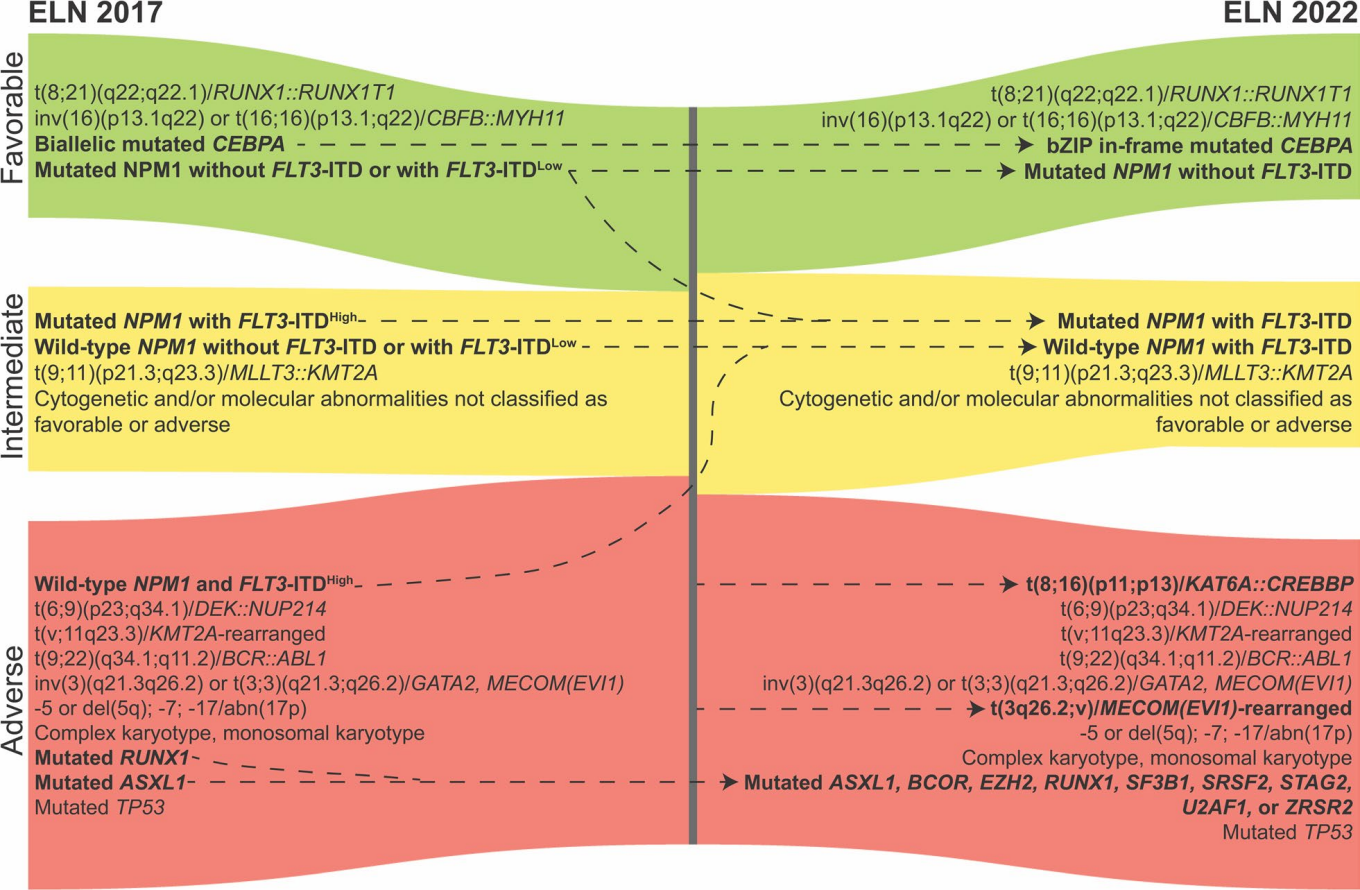
Updates in ELN risk stratification of AML. A Sankey plot depicts changes in the 2017 [65] and 2022 [35] ELN risk stratification of AML. Prognostic groups are groups by color (favorable—green, intermediate—yellow, adverse—red) and changes are tracked by dashed arrows. *bZIP* Basic leucine zipper domain, *ELN* European LeukemiaNet

These changes reflect a movement toward molecularly defining AML and related myeloid neoplasms. The natural history of these diseases suggests some overlap in pathogenesis with biological variability perhaps better attributed to distinct genetic driver events rather than morphologic differences. Thus, the European LeukemiaNet (ELN) also updated their risk stratification schema (Fig. 3) to reflect this change [35]. The favorable prognostic impact of *CEBPA* mutations is driven by bZIP domain mutations [29–31], so this is now specified in the favorable-risk category. Moreover, *FLT3* internal tandem duplication (*FLT3-*ITD) mutations had previously been considered a VAF-dependent risk factor in patients who also harbor *NPM1* mutations. However, there is variability in the standardization of measuring allelic ratio (AR), and the incorporation of *FLT3*-targeting multi-tyrosine kinase inhibitor (TKI) midostaurin (MIDO) has demonstrated benefit in patients with *FLT3*-ITD regardless of VAF [36]. Thus, for epidemiologic and practical reasons, *FLT3*-ITD is now considered intermediate-risk irrespective of AR or co-occurring *NPM1* mutations. Although patients with *FLT3*-ITD who did not receive MIDO had worse outcomes regardless of ELN 2017 risk category [36], MRD testing could be considered for more dynamic risk stratification after induction therapy and/or prior to alloHSCT given its prognostic relevance even for patients treated with chemotherapy only [37, 38]. In concordance with the expanded list of myelodysplasia-associated genes from the WHO and ICC, mutations in *ASXL1*, *BCOR*, *EZH2, RUNX1*, *SF3B1, SRSF2*, *STAG2*, *U2AF1*, or *ZRSR2* are now considered adverse-risk based on updated prognostic studies in MDS, de novo AML, and secondary AML (sAML) [25–28, 39, 40]. Lastly, new disease-defining cytogenetic changes involving *MECOM *[41, 42] or *KAT6A::CREBBP* fusion [43] have been updated in adverse-risk disease, while hyperdiploid karyotype with multiple trisomies/polysomies is no longer considered complex karyotype. The changes to the ELN risk stratification system reflect an evolving understanding of how AML biology impacts clinical phenotype. However, genetic drivers of AML do not always appear in isolation [16, 44], and, as we will discuss later in this review, co-mutation patterns often have conflicting prognostic implications. Accordingly, a more comprehensive incorporation of genetic and cytogenetic alterations along patient and disease characteristics, as has recently been implemented in MDS [40], may improve that personalization of risk stratification.

## Azacitidine and venetoclax: moving up the ranks

### AML in those unfit for intensive chemotherapy

It should be noted that while updates from the WHO, ICC, and ELN emphasize the importance of molecular pathogenesis in AML, there are other patient characteristics that significantly impact prognosis and treatment, namely age and fitness. The National Comprehensive Cancer Network (NCCN) guidelines divide induction treatment algorithms based on age below or above 60 years [45]. While the age of 60 is not uniform in its capacity to discriminate the ability to tolerate high-intensity chemotherapy, clinical trials have traditionally been organized around this age. However, in clinical practice, these historical age cutoffs warrant reconsideration when patients are physiologically fit despite being chronologically older. Historically, non-intensive therapy included the use of single-agent azacitidine (AZA) or decitabine (DAC) with a median OS reaching a dismal 7.7 to 10.4 months [46, 47]. The combination of the BCL2-inhibitor venetoclax (VEN) with AZA or low-dose cytarabine (LDAC) has changed the standard of care for this patient population. The VIALE-A study [48] compared AZA/VEN to AZA/placebo in an elderly population (median age 76 years) and observed an improvement in the rate of complete remission (CR) plus CR with incomplete hematologic recovery (CRi) (66.4% versus 28.3%, respectively) and median OS (14.7 months versus 9.6 months, respectively). Long-term follow-up from this study confirms these findings and notably observed that patients who achieved CR/CRi and MRD negativity with AZA/VEN had a median OS of 34.2 months [49]. Similarly, the VIALE-C study [50] randomized patients with a median age of 76 years to LDAC/VEN or LDAC/placebo, noting an improvement in the rate of CR/CRi from 13 to 48% along with survival benefit at long-term follow-up [51, 52]. Recently, a study by Pollyea et al. found that the use of alloHSCT in patients over 60 years who received AZA/VEN improved median OS compared to those who deferred alloHSCT (not reached versus 17.2 months) [53]. Another retrospective study from the same group [54] compared outcomes of patients undergoing alloHSCT after AZA/VEN to intensive chemotherapy and found no significant difference in recurrence-free survival (RFS) (73.2% versus 66.1%, respectively) or OS (76.3% versus 74.7%, respectively) at 12 months. Age was not a predictive factor of death or relapse in this population, though higher hematopoietic cell transplantation-specific comorbidity index (HCT-CI) and positive pre-alloHSCT MRD were associated with worse outcomes. Theoretically, in older patients with high-risk disease, treatment with AZA/VEN may maintain or improve fitness, allowing more patients to undergo alloHSCT who may not have previously been eligible after receiving induction with 7 + 3. Currently, there is a study (NCT04801797) ongoing to address this question which randomizes patients fit for intensive chemotherapy to receive either 7 + 3 or AZA/VEN.

### Secondary AML

As previously discussed, most cases of AML arise de novo, but sAML presents a unique clinical challenge. While the incidence of sAML increases with age [55], sAML frequently harbors adverse-risk cytogenetics and mutational profiles that are often associated with treatment resistance [56–60]. CPX-351, a fixed 5:1 molar ratio of cytarabine/daunorubicin liposome, was developed with the intention of optimizing a synergistic molar ratio of the two chemotherapeutics [61] and was noted to have activity in sAML in early data [62]. A subsequent phase 3 study assessed CPX-351 versus 7 + 3 in patients aged 60–75 with newly diagnosed (ND) tAML, antecedent MDS or chronic myelomonocytic leukemia, or de novo AML with MDS-related cytogenetics (AML-MRC) [63] and observed improved median OS (9.56 months versus 5.95 months) and CR/CRi rates (47.7% versus 33.3%), leading to FDA approval of CPX-351 for patients with newly ND tAML or AML-MRC. Moreover, long-term survival with CPX-351 was attributed to more patients proceeding to alloHSCT, which was performed in 34% of patients [63].

In clinical practice, there is significant overlap in patients who may be eligible for AZA/VEN or CPX-351 in the frontline setting, though these two regimens have not been compared head-to-head. A recent real-world analysis of patients with ND AML who received AZA/VEN or CPX-351 found that those receiving AZA/VEN were more likely to be older (median age 75 years versus 67 years, respectively) [64]. Despite the heterogeneity between cohorts, median OS for all patients was similar between the AZA/VEN and CPX-351 cohorts with no significant difference even when controlling for multiple factors including performance status, MRC, ELN 2017 risk category [65], high-risk mutations, and HCT-CI score. Although fewer patients underwent transplant with AZA/VEN induction, median OS was not significantly affected by the choice of CPX-351 versus AZA/VEN. Thus, while a randomized control trial is lacking, this study provides some clinical equipoise between AZA/VEN and CPX-351 in patients who may be eligible for either therapy.

### AML with high-risk mutations

With advances in next-generation sequencing (NGS) technology, the detection of molecular driver mutations in AML has improved. Follow-up analyses of several landmark studies have allowed for the identification of certain mutational subgroups which may derive further benefit from AZA/VEN compared to intensive chemotherapy. Perhaps the most challenging scenario is with *TP53*-mutant AML. Recent data suggest that patients with *TP53* mutations have worse outcomes compared to other adverse-risk features with a 2-year OS of only 12.8% even with intensive treatment [33]. The presence of *TP53* mutations confers similarly poor outcomes irrespective of de novo disease, sAML, or blast count [34]. However, there is heterogeneity in *TP53*-mutant AML based on types of mutations, involved domains, and VAF; a recent report found that patients without abnormal p53 protein expression, intact copy number, and low VAF had a more favorable prognosis in the spectrum of *TP53*-mutant AML [66]. Nonetheless, even in patients who undergo alloHSCT, relapse rates are very high with median OS often reported as less than 6 months after transplant [67, 68], though this may be longer based on more recent retrospective studies [69]. In the VIALE-A study, CR/CRi for patients with *TP53* alterations was drastically improved with AZA/VEN (55.3%) compared to AZA/placebo (0%), though the duration of remission (DOR) was brief, and median OS was only 7.2 months [48, 70].

Notably, AZA/VEN achieved CR/CRi rates of 70% compared to 23% with AZA alone in patients with adverse-risk cytogenetics without *TP53* mutations, along with durable remissions (18.4 months versus 8.51 months) and improved median OS (23.4 months versus 11.3 months). However, the benefit in DOR or OS was lost with the addition of *TP53* mutations despite improvements in CR/CRi (41% with AZA/VEN versus 17% with AZA) [71]. Unfortunately, several follow-up studies with hypomethylating agents (HMA) and VEN have produced similarly discouraging results [71–73]. The lack of durable response may stem from a requirement of intact p53 protein to maintain long-term response to BH3-mimetic drugs such as VEN [74]. While HMA/VEN-based regimens have not yet demonstrably moved the needle forward for patients with *TP53*-mutant AML, several studies are underway combining these agents in this high-risk disease; these will be discussed later in this review.

Two other high-risk gene mutations of interest in the context of AZA/VEN are *ASXL1* and *RUNX1*. Preclinical data from isogenic leukemic cells harboring *ASXL1* mutations demonstrated that genetic correction of this variant slows leukemic cell growth and induces differentiation [75]. CD34 cells with *ASXL1* mutations from patients were also shown to have higher *BCL2* expression and gene-body methylation, rendering them more sensitive to VEN and AZA, respectively [75]. These findings appear clinically relevant as retrospective studies in patients with R/R AML [76] and MDS-EB2 [77] treated with HMA/VEN observed improved CR/CRi rates in patients with *ASXL1* mutations. In the latter study, patients harboring *ASXL1* mutations were found to have a better median OS (not reached) compared to those without (10.2 months). However, an independent study could not reproduce improved CR/CRi rates in patients with *ASXL1* mutations treated with AZA/VEN compared to intensive chemotherapy [78]. Rather, they observed improved CR/CRi rates in patients with *RUNX1* mutations when using AZA/VEN compared to intensive chemotherapy. A significant survival benefit was observed in older patients with *RUNX1* mutations receiving AZA/VEN as well. Although retrospective in nature, these data suggest a preferential benefit of HMA/VEN in patients with *RUNX1* and *ASXL1* mutations and highlight the importance of mutational testing in treatment planning.

## Targeted oral therapy combinations

The last 5 years has led to multiple targeted therapy approvals for patients with AML and mutations in *IDH1* (ivosidenib [IVO], olutasidenib [OLU]), *IDH2* (enasidenib [ENA]), and *FLT3* (MIDO, gilteritinib [GILT]). We will briefly review the landmark studies regarding these agents and discuss the emerging roles for approved therapies (Table 2) as well as ongoing areas of investigation into their use.

**Table 2:** Published studies of approved and investigational uses of FDA-approved and/or NCCN-recommended targeted therapies in AML

Targeted therapy	AML study indication (Ref.)	Study arms	Median age (years)	N	CR (%)	CRc (definition, %)	mOS (months)	Final dose/schedule in study	FDA-approved use	NCCN-recommended use
IVO	R/R [87]	IVO, single arm	67	125	21.6	CR/CRh, 30.4	8.8	500 mg daily	1. Frontline monotherapy for ND *IDH1*-AML if ≥ 75 years or unfit for IC 2. Frontline IVO/AZA for ND *IDH1*-AML if ≥ 75 years or unfit for IC 2. Monotherapy for R/R *IDH1-*AML	1. Frontline monotherapy for *IDH1*-AML if ≥ 60 years and unfit for IC 2. Frontline IVO/AZA for *IDH1*-AML if ≥ 60 years old and unfit for IC 3. Maintenance monotherapy after low-intensity induction for *IDH1*-AML 4. Monotherapy for R/R *IDH1-*AML
	ND [88]	IVO, single arm	76.5	34	30.3	CR/CRh, 42.4	12.6			
	ND [93]	IC/IVO, single arm	62.5	60	68.3	CR/CRi/CRp, 76.7	NR Estimated 12-month OS 78%	500 mg daily starting day 1 of IC ± consolidation ± maintenance		
	ND [97]	AZA/IVO	76	23	60.9	CR/CRi/CRp, 69.6	NR Estimated 12-month OS 82.0%	IVO 500 mg daily (day 1–28) AZA 75 mg/m^2^ daily (day 1–7)		
	ND [98]	AZA/IVO vs. AZA/PBO	76 vs. 75.5	72 vs. 74	47.2 vs. 14.9	CR/CRi/CRp, 54.2 vs. 16.2	24.0 vs. 7.9			
OLU	R/R [91]	OLU vs. AZA/OLU	72 vs. 67	22 vs. 26	18 vs. 12	CR/CRp, 32 vs. 15	8.7 vs. 12.1	OLU 150 mg twice daily AZA 75 mg/m^2^ daily (day 1–7)	1. Monotherapy for *IDH1*-AML	None
	ND [91]	OLU vs. AZA/OLU	72 vs. 67	4 vs. 13	0 vs. 54	CR/CRp, 0 vs. 54	8.8 vs. NR			
ENA	R/R [82, 83]	ENA, single arm	68	214	19.6	CR/CRi/CRp, 29.0	8.8	100 mg daily	1. Monotherapy for R/R *IDH2-*AML	1. Frontline monotherapy for *IDH2*-AML if ≥ 60 years and unfit for IC 2. Maintenance monotherapy after low-intensity induction for *IDH2*-AML 3. Monotherapy for R/R *IDH2-*AML
	R/R [85]	ENA vs. Conventional care	72 vs. 71	158 vs. 161	23.4 vs. 3.7	CR/CRi/CRp, 29.7 vs. 6.2	6.5 vs. 6.2			
	R/R [100]	AZA/ENA, single arm	64	19	27.7	CR/CRi, 61.1	Not reported Estimated 12-month OS if treated in 1st relapse 75% vs. ≥ 2nd relapse 10%	ENA 100 mg daily (day 1–28) AZA 75 mg/m^2^ daily (day 1–7)		
	ND [86]	ENA, single arm	77	39	17.9	CR/CRi/CRp, 20.5	11.3	100 mg daily		
	ND [93]	IC/ENA, single arm	63	93	54.9	CR/CRi/CRp, 73.6	25.6	100 mg daily starting day 1 or day 8 of IC		
	ND [101]	AZA/ENA vs. AZA/PBO	75 vs. 75	68 vs. 33	54.4 vs. 12.1	CR/CRi/CRp, 63.2 vs. 30.3	22.0/22.3	ENA 100 mg daily (day 1–28) AZA 75 mg/m^2^ daily (day 1–7)		
	ND [100]	AZA/ENA, single arm	77	7	71.4	CR/CRi, 100	NR Estimated 12-month OS for ND group was 83%			
MIDO	ND [114]	IC/MIDO, single arm	Total (48.5) *FLT3* mutant [46]	Total [40] *FLT3* mutant [13]	Total [80] *FLT3* mutant [92]	Not reported	Not reported Estimated 12-month OS for *FLT3*-mutant 85%	50 mg BID on days 1–7 and days 14–21 (concomitant) or days 8–21 (sequential) of IC ± consolidation ± maintenance	1. MIDO + IC, re-induction, and consolidation for *FLT3-*TKD or ITD AML if eligible for IC	1. MIDO + IC, re-induction, and consolidation for *FLT3-*TKD or ITD AML if eligible for IC and with intermediate/poor-risk cytogenetics
	ND [118]	IC/MIDO vs. IC	47.1 vs. 48.6	360 vs. 357	58.9 vs. 53.5	Not reported	74.7 vs. 25.6	50 mg BID on days 8–21 of IC ± consolidation ± maintenance		
	ND [121, 122]	IC/MIDO, single arm	54.1	440	37	CR/CRi, 74.9	36.2	50 mg BID on days 8 of IC continuously until 48 h prior to next cycle ± consolidation ± maintenance		
GILT	R/R [117]	GILT vs. Conventional care	62.0 vs. 61.5	247 vs. 124	21.1 vs. 10.5	CR/CRi/CRp, 54.3 vs. 24.8	9.3 vs. 5.6	120 mg daily	1. GILT monotherapy for R/R *FLT3*-TKD or ITD AML	1. GILT monotherapy for R/R *FLT3*-TKD or ITD AML
	R/R [135]	VEN/GILT, single arm	63	56	18	CR/CRi/CRp, 39	10.0	GILT 120 mg daily VEN 400 mg daily		
SORA	R/R [130]	AZA/SORA, single arm	64	43	16	CR/CRi, 43	6.2	SORA 400 mg BID (days 1–28) AZA 75 mg/m^2^ daily (day 1–7)	None	1. SORA + AZA or DAC + for R/R *FLT3-*ITD AML 2. Frontline SORA + AZA or DAC for *FLT3-*ITD AML if unfit for IC 3. SORA + AZA or DAC + SORA maintenance after low-intensity induction for *FLT3*-ITD AML 4. SORA maintenance after alloHSCT in *FLT3*-ITD AML
	ND [136]	AZA/SORA, single arm	74	27	26	CR/CRi/CRp, 70	8.3			
	ND [144, 145]	IC/SORA vs. IC	50 vs. 50	134 vs. 133	60 vs. 59	Not reported	NR vs. NR 5-year OS 61% vs. 53%	400 mg BID on days 10–19 of IC ± consolidation ± maintenance		
	MAIN [146]	SORA vs PBO	54.2 vs. 53.6	43 vs. 40	N/a	N/a	NR vs. NR Estimated 2-year OS 90.5% vs. 66.2%	400 mg BID		
	MAIN [148]	SORA vs PBO	35 vs. 35	100 vs. 102	N/a	N/a	NR vs. NR Estimated 2-year OS 82.1 vs. 68.0			

*CR* Complete remission, *CRc* Composite complete remission, *CRi* Complete remission with incomplete hematologic recovery, *CRp* Complete remission with incomplete platelet recovery, *CRh* Complete response with partial hematologic recovery, *mOS* Median overall survival, *FDA* Food and Drug Administration, *NCCN* National Comprehensive Cancer Network, *R/R* Relapsed/refractory, *ND* Newly diagnosed, *MAIN* Maintenance, *alloHSCT* Allogeneic stem cell transplant, *IVO* Ivosidenib, *OLU* Olutasidenib, *ENA* Enasidenib, *MIDO* Midostaurin, *GILT* Gilteritinib, *SORA* Sorafenib, *AZA* Azacitidine, *VEN* Venetoclax, *IC* Intensive induction chemotherapy, *PBO* Placebo, *NR* Not reached, *BID* Twice daily

### *IDH1* and *IDH2*-mutant AML

*IDH1* and *IDH2* mutations are reported at a frequency of 7–14% and 8–19%, respectively, in AML [16, 79]. Mutations in these genes typically occur in the conserved arginine residues (IDH1^R132^, IDH2^R140^, and IDH2^R172^) of the catalytic domain of isocitrate dehydrogenase. The prognostic implications of *IDH1/IDH2* mutations are not entirely clear. Some reports have suggested that *IDH2* mutations are associated with better outcomes while *IDH1* mutations confer worse outcomes [80, 81], though there is significant heterogeneity in the prognostic impact of co-mutations such as *NPM1* [16, 81].

In a phase 1/2 study, patients with R/R AML harboring IDH2^R140^ or IDH2^R172^ mutations (mean age 67 years) were treated with ENA [82, 83]. Patients with IDH2^R140^ mutations had 2-HG reductions greater than 90% regardless of response, while 2-HG levels in those with IDH2^R172^ mutations correlated with response (82.0% reduction from baseline if CR, 44.3% reduction from baseline if non-CR response, and 38.4% reduction from baseline if no response). CR/CRi/CR with incomplete platelet recovery (CRp) was observed in 29% of patients. Median OS for all patients was 8.8 months but extended to 22.9 months in patients achieving CR. While reduction in mutant *IDH2* VAF was not required for response, follow-up data demonstrated that clearance of *IDH2-*mutated clones was associated with 100% CR [83]. Furthermore, co-occurring mutations in *NRAS* or MAPK pathway were suggested to contribute to treatment resistance [84]. In a phase 3 randomized study from the BEAT AML Master trial, older patients with *IDH2-*mutant R/R AML were randomized to ENA or conventional care [85]; patients in the ENA arm had a doubling of event-free survival (EFS) and significant improvements in CR/CRi/CRp rates and hematologic response. The presence of *DNMT3A* co-mutations has been shown to be associated with CR, and no deleterious effects of RAS signaling pathway co-mutations were observed; however, the presence of ≥ 4 co-mutations decreased the overall response rates (ORR) significantly (27.3% compared to 47.1% with < 4 co-mutations) [86]. Thus, although ENA only has an FDA-approval label for R/R AML with *IDH2* mutations, the NCCN guidelines provide a recommendation to consider frontline ENA use for patients older than 60 years who are not candidates for intensive remission induction [45].

In a phase 1 study, DiNardo et al. assessed the use of IVO in *IDH1-*mutant R/R AML [87]. The median age of patients was 67 years, and CR/CRp rates were 30.4% with a median DOR of 8.2 months. Median OS was 8.8 months with an 18-month OS rate of 50.1% in patients with CR/CRp. It was noted that patients with a lower co-mutational burden had improved CR/CRp rates, but no specific predictive co-mutations were identified. As a follow-up to this study, 34 patients with ND AML and *IDH1* mutations (median age 76.5 years) received IVO in the frontline setting [88]. The composite CR (CRc) rate was 42.4% with over 60% of patients maintaining CRc at 1 year. Patients who received prior HMA therapy for antecedent hematologic disorder achieved CRc approximately half as frequently as those without prior HMA. Receptor tyrosine kinase (RTK) pathway mutations were observed in 36.8% of patients who did not achieve CRc compared to no patients who achieved CRc. *IDH1* mutant clone clearance was reported in 64.3% of patients who achieved CRc and was not observed in any patients without CRc. These two studies ultimately led to the approval of IVO in both R/R AML with *IDH1* mutations and ND AML with *IDH1* mutations in patients who are ineligible for standard chemotherapy.

On December 1, 2022, the FDA approved another IDH1 inhibitor, OLU [89], for use in R/R AML based on a phase 1/2 trial of patients with *IDH1*-mutant R/R AML who were naïve to IDH1 inhibitors [90]. Patients with a median age of 71 years were treated with OLU until progression. Notably, CR/CRh rates were 35% and were achieved at a median of 1.9 months. Responses appeared durable with a median DOR of 25.9 months in patients who achieved CR/CRh. Prior VEN exposure did not appear to decrease response efficacy. Survival data will require further maturation. Another phase 1/2 study examining OLU with or without AZA in ND and R/R AML demonstrated similar CR/CRh rates in R/R AML patients treated with monotherapy while responses were surprisingly worse when combined with AZA [91]. While the role of OLU is not clear considering experience with using IVO, one compelling scenario could be in the setting of IVO resistance through *IDH1* mutations, though this is solely based on preclinical data [92] with no current data on response rates after prior IVO exposure.

Recent studies have evaluated the use of IVO or ENA in combination with other frontline therapies in ND AML. A phase 1 study assessed the use of IVO or ENA in combination with 7 + 3 (or bioequivalent dose of idarubicin) in patients with *IDH1/IDH2* mutations [93]. Patients received IVO or ENA throughout induction, consolidation, and maintenance, though IVO or ENA were discontinued in patients who underwent alloHSCT. CR/CRi/CRp rates were 72% for IVO and 63% for ENA at the end of induction, which is slightly better compared to historical controls with *IDH1/2* mutations [94]. In an updated analysis [95], the authors reported CR/CRi/CRp rates of 78.3% in the IVO subgroup and 73.6% in the ENA subgroup. Responses for the sAML subgroup were improved if there was no prior HMA exposure, consistent with previously reported data [87]. Co-mutations did not impact response rates in the IVO cohort, but in the ENA cohort, co-mutations with *ASXL1, NRAS, U2AF1,* and *TP53* were associated with worse response rates, while *DNMT3A* co-mutations were associated with marginally improved CR/CRi/CRp. When assayed by digital polymerase chain reaction (dPCR), 39% of patients treated with IVO cleared *IDH1*-mutant clones and 23% of patients treated with ENA cleared *IDH2*-mutant clones. Approximately half of the patients receiving IVO or ENA ultimately proceeded to alloHSCT. The use of IVO or ENA in combination with induction therapy was tolerable and ultimately did not significantly impact the time to recovery of the absolute neutrophil count or platelet count. An unanswered question that will require further investigation is the role of maintenance IVO or ENA after alloHSCT, particularly in patients who are unable to clear their mutant clone prior to transplantation. In patients eligible for intensive chemotherapy, the improved response rates compared to historical controls with the addition of IVO or ENA [93, 95] provides a compelling argument for this practice. However, in the absence of randomized head-to-head comparison, this combination is neither FDA-approved nor recommended by the NCCN guidelines [45].

The majority of *IDH1/2* mutations have been shown to be exquisitely sensitive to the combination of AZA/VEN with response rates similar to or higher than those achieved with standard induction. Therefore, it is reasonable to consider low-intensity therapy in this patient population without compromising outcomes. In a pooled analysis of from VIALE-A [48] and a phase 1b HMA/VEN study [70], the patients with *IDH1/2* mutations achieved CR/CRi rates of 79% with AZA/VEN compared with 11% with AZA alone [96]. Median DOR was 29.5 months and 9.5 months, respectively, and median OS was 24.5 months and 6.2 months, respectively. CR/CRi and OS were relatively better with *IDH2* mutations compared to *IDH1*.

The combination of AZA with IVO has recently emerged as an alternative non-intensive treatment option [97]. The AGILE study was a phase 3 trial randomizing patients with ND *IDH1*-mutant AML ineligible for intensive chemotherapy to receive AZA/IVO or AZA/placebo [98]. At a median follow-up of 12.4 months, EFS was significantly longer in the AZA/IVO group compared to AZA/placebo with an estimated 12-month EFS of 37% and 12%, respectively. As a secondary endpoint, the median OS was 24 months for AZA/IVO and 7.9 months for AZA/placebo. CR/CRp rates were 53% with AZA/IVO compared to 18% with AZA/placebo, and DOR was longer for AZA/IVO compared to AZA/placebo (22.1 months versus 9.2 months). Patients with RTK pathway mutations (*FLT3, KIT, NRAS, KRAS, PTPN11*) and *TP53* mutations were more likely to respond to AZA/IVO, and follow-up data suggest that relapse appears to preferentially occur with the acquisition of secondary high-risk mutations, independent of *IDH1 *[99]. The findings of this study led to the recent FDA approval of AZA/IVO for the frontline treatment of patients with ND *IDH1-*mutant AML. While the rate of differentiation syndrome in these patients approaches 20%, the rate of cytopenias compared to AZA/VEN is significantly less. Therefore, when thinking about the various options for patients with *IDH1* mutations, toxicity, quality of life, and sequencing of treatment should be considered.

The combination of AZA/ENA has also been studied in patients with R/R [100] and ND AML with *IDH2* mutations ineligible for intensive chemotherapy [100, 101]. In the phase 2 analysis of patients with ND AML, patients were randomized to AZA/ENA or AZA monotherapy [101]. The median age of patients was 75 years with CR/CRi/CRp rates of 63% with the AZA/ENA group compared to 30% with AZA alone; similar ORR were seen regardless of R140 or R172 mutations. Both ORR and CR were more durable with AZA/ENA compared to AZA (24.1 months versus 9.9 months and not reached versus 12.7 months, respectively). In a 2-year post hoc analysis, median EFS with AZA/ENA was 15.7 months compared to 11.9 months with AZA alone and OS was 22 months with AZA/ENA compared to 18.6 months with AZA alone; while the survival differences were not statistically significant, this study was not powered to detect significant differences in survival outcomes.

A major question remains as to whether therapies such as AZA/IVO or AZA/ENA would outperform AZA/VEN for ND *IDH1/2*-mutant AML. The lack of survival advantage with AZA/ENA compared to AZA monotherapy [101] would suggest that AZA/VEN is superior in *IDH2* mutations with the caveat that the median OS of patients with AZA in this study was significantly longer than reported for patients with *IDH2* mutations in the AZA/VEN studies [96]. For patients with *IDH1* mutations, CR rates appear to be better with AZA/VEN [96] than with AZA/IVO [97], though median OS was essentially the same. At present, the widespread availability of AZA/VEN and its ability to bridge to alloHSCT favors its use in the frontline setting for patients with *IDH1/2* mutations who are ineligible for intensive chemotherapy, thereby preserving IVO, OLU, or ENA in the case of R/R disease. However, AZA/IVO could be considered in patients who are at high risk of complications with the myelosuppression of AZA/VEN. While ENA and IVO monotherapy are both NCCN-recommended options for frontline therapy [45], only IVO is approved in this setting, and these should only be considered in patients with a very poor performance status. An area for future study will be triplet therapies. In an exploratory study of patients with treatment-naïve and R/R AML with *IDH1* mutations [102], patients who received AZA/VEN/IVO had a CRc rate of 85–100% depending on dose intensity; however, this compares similarly to CRc rates of 67–100% with IVO/VEN alone. Nevertheless, the use of a triplet regimen improved MRD to 86% from 25% with doublet therapy. Studies combining different permutations of ENA, OLU, or IVO with HMA and/or VEN are currently underway (NCT04092179, NCT03471260, NCT04774393, NCT02719574) as are studies of these agents in the maintenance setting (NCT05010772, NCT03728335, NCT03564821, NCT03515512, NCT04522895).

### *FLT3*-mutant AML

*FLT3* encodes a type 3 RTK (FMS-like tyrosine kinase 3) and is widely expressed on AML blasts [103]. Mutations in *FLT3* are seen in about 25–32% of cases of ND AML with 25% harboring ITDs and 7–10% harboring TKD mutations [104, 105]. Previous reports prior to the era of *FLT3*-targeting TKIs (henceforth referred to as FLT3i) have suggested that AR of *FLT3*-ITD and the presence of *NPM1* co-mutations variably affect outcomes [106–108]. Nevertheless, recent data suggest that relapse risk is higher in patients with *FLT3*-ITD AML irrespective of AR or presence of *NPM1* mutation, and these patients should be considered for alloHSCT in first remission (CR1) if eligible [109, 110]; this is reflected in the updated ELN recommendations [24]. FLT3i can be divided into Type I and Type II inhibitors [111], which are active against both ITD and TKD mutations or ITD only, respectively. The first approved FLT3i in AML was the Type I staurosporine-derived inhibitor MIDO [112, 113] with early reports of its synergy with chemotherapy in patients with ND *FLT3*-mutant AML [114]. Newer and more selective FLT3i, GILT (Type I) and quizartinib (QUIZ) (Type II), have demonstrated promising responses in patients with R/R AML [115–117].

The RATIFY trial [118] was a randomized, placebo-controlled phase 3 trial investigating the addition of MIDO to standard induction chemotherapy and high-dose cytarabine (HiDAC) consolidation in adult patients under the age of 60 with ND AML and *FLT3* mutations (TKD or ITD). CR rates were similar between both groups, though median EFS and OS were significantly improved with MIDO (8.2 months versus 3 months and 74.7 months versus 25.6 months, respectively). OS was durable with 51.4% of patients in the MIDO group surviving at 4 years. More patients underwent alloHSCT in CR1 with MIDO compared to placebo (28% versus 23%, respectively); notably, follow-up studies after the publication of the RATIFY trial demonstrated deeper molecular remission with the addition of FLT3i to induction therapy [119, 120], perhaps explaining in part the durable differences in OS despite similar CR and EFS rates.

Recent findings from a phase 2 study have also established the efficacy of adding to MIDO to induction, HiDAC consolidation, and maintenance in patients up to the age of 70 with ND *FLT3*-mutant AML [121, 122]. It should be noted that at present, MIDO is not currently approved as monotherapy and therefore is not recommended for post-consolidation maintenance given minimal benefit demonstrated after alloHSCT [36, 118, 121–123]. Preliminary data from a phase 1 study (NCT02236013) evaluating GILT in combination with 7 + 3, consolidation, and maintenance in ND AML [124] noted a median OS of 35.8 months with CRc achieved by 81.8% of all patients. AlloHSCT was performed in 30.4% of all patients. These data have led to ongoing clinical trials of GILT versus MIDO in addition to induction chemotherapy and consolidation (NCT04027309, NCT03836209).

GILT is the only FDA-approved FLT3i for use in R/R AML with *FLT3* mutations. The ADMIRAL study was a phase 3 randomized control trial of patients with R/R AML and *FLT3*-mutations who received GILT or salvage chemotherapy [117]. Similar rates of prior FLT3i exposure were noted in both arms, and approximately 20% of patients in either group had previously undergone alloHSCT. Median OS for patients receiving GILT was 9.3 months versus 5.6 months for those receiving salvage chemotherapy. [125]. CRc rates were 54.3% with GILT and 24.8% with chemotherapy, and the median DOR was 11 months in the GILT group. Median OS for the *FLT3-*ITD and *FLT3*-TKD groups that received GILT were 9.3 months and 8 months, respectively. An important aspect of this study was the efficacy in both *FLT3*-ITD and *FLT3*-TKD populations, as the latter has been demonstrated to confer secondary resistance to type II FLT3i [126].

Given the increasing use of low-intensity regimens in AML, pooled data from VIALE-A [48] and a phase 1b HMA/VEN study [70] showed that patients with *FLT3-*ITD had a CR/CRi rate of 63% with AZA/VEN and a median OS of 9.9 months, while those with *FLT3-*TKD had a CR/CRi rate of 77% and a median OS of 19.2 months [127]. Of patients with *FLT3* mutations, approximately 36% had *NPM1* mutations in each of the AZA/VEN and AZA groups. Of those with concurrent *FLT3-*ITD and *NPM1* mutation, AZA/VEN conferred a CR/CRi rate of 70% and a median OS of 9.1 months; patients with *FLT3*-ITD and wild-type *NPM1* had a median OS of 10.6 months. This study has two important takeaways for older patients with mutated *FLT3*. First, the rate of *FLT3* mutations in this population was lower, and the patients were older than typical *FLT3*-driven AML seen in younger patients, possibly suggesting different disease kinetics and biology. Second, while CR/CRi rates were worse for patients with *FLT3*-ITD mutations with wild-type *NPM1* compared to those with mutated *NPM1*, the overall survival did not differ significantly, suggesting that *NPM1* status has an unclear prognostic value for patients treated with HMA/VEN.

Trials combining GILT [128] or MIDO [129] with HMA have not yielded encouraging results to date, though early data suggest that AZA/sorafenib (SORA) may be effective in patients with R/R AML and *FLT3*-ITD [130]. Data demonstrating the efficacy and tolerability of DAC/SORA in patients with R/R AML with *FLT3*-ITD [131] have led to NCCN recommendations for the use of AZA/SORA or DAC/SORA as low-intensity therapy in elderly patients with *FLT3*-ITD AML or in R/R AML with *FLT3-*ITD [45], though it does not carry FDA approval for these indications. Despite the modest benefit with HMA, there seems to be synergy between FLT3i and VEN [132–134]. A phase 1b study for VEN/GILT enrolled patients with *FLT3*-wild-type or *FLT3-*mutant (dose escalation) and *FLT3-*mutant (dose expansion) R/R AML [135]. The median age of patients was 63 years, 31% of whom had received prior alloHSCT and 16% of whom received prior VEN. No patients had previously received GILT, though 64% of patients with *FLT3* mutations had received other prior FLT3i. Patients with *FLT3*-ITD had CR/CRi/CRp rates of 43%, while those with *FLT3-*TKD has rates of 33%, and response rates were slightly better in those who were FLT3i-naïve. Median OS was 10 months for all *FLT3*-mutated patients, though there was a significant improvement in those who had undergone alloHSCT after VEN/GILT (not reached) compared to those who did not receive alloHSCT (6.3 months).

Furthermore, a phase 2 trial evaluated the use of triplet therapy (DAC/VEN/FLT3i) in older patients with ND *FLT3*-mutant AML and all adult patients with R/R *FLT3-*mutant AML. In ND AML, the CRc rate was 92% with high rates of 91% MRD negativity in responders by PCR. In patients with R/R AML, CRc rates were 63% with MRD negativity by PCR in all patients who responded. At a median follow-up of 14.5 months, the median OS was not reached in ND patients (2-year OS estimated at 80%); the median OS in R/R patients was 6.8 months. Approximately one-third of patients underwent alloHSCT in either group. These results compare favorably to other reports of FLT3i/HMA in the ND setting [128, 131, 136], though CRc rates appear to be higher with VEN/GILT in patients with R/R AML [135].

Although not approved, two other FLT3i deserve mention given recent reports of their efficacy in AML. QUIZ is a second-generation type I FLT3i that can achieve significant marrow remissions in R/R *FLT3-*mutant AML [116, 137–139], though survival advantage was minimal compared to salvage chemotherapy in the phase 3 QuANTUM-R study [138]. Due to these underwhelming results and concerns about cardiotoxicity and increased myelosuppression compared to other FLT3i, QUIZ has not been approved in the USA or Europe, though it is approved for use in Japan. In the frontline setting, the phase 3 QuANTUM-FIRST (NCT02668653) trial [140] enrolled patients up to age 75 with ND AML and *FLT3*-ITD and randomized them to QUIZ or placebo in addition to induction therapy with 7 + 3. Patients who achieved CR/CRi received up to 4 cycles of HiDAC with QUIZ or placebo and/or alloHSCT followed by up to 3 years of maintenance therapy with QUIZ or placebo. CR/CRi rates were 71.6% and 64.9% in the QUIZ and placebo arms, respectively, with DOR of 38.6 months and 12.4 months, respectively. Median OS and RFS were 31.9 months versus 15.1 months and 39.3 months versus 13.6 months in the QUIZ and placebo arms, respectively. AlloHSCT was performed in CR1 at similar rates between both arms; when censored for alloHSCT, OS trended toward a benefit with QUIZ over placebo. Moreover, an updated report from the study found that QUIZ conferred a deeper molecular remission compared to the placebo arm, perhaps underscoring the durability of benefit [141]. Although RATIFY [118] had already demonstrated a benefit to the addition of MIDO to induction chemotherapy, the QuANTUM-FIRST study is unique in that it evaluates the addition of an FLT3i for the higher-risk *FLT3*-ITD mutation.

Lastly, a type I FLT3i emerging in clinical discussion is crenolanib (CREN). Long-term data were recently reported regarding the use of CREN in combination with 7 + 3 in adult patients with *FLT-*mutant ND AML [142]. CREN maintenance was offered up to 1 year after HiDAC or alloHSCT. The median age of patients enrolled was 57 years, 34% of which were over the age of 60 years. *FLT3* mutations were 75% ITD, 18% TKD, and 7% both ITD and TKD. CR/CRi rates above 80% were reported across several subgroups including those with *FLT3*-ITD mutations or concomitant *FLT3/DNMT3A/NPM1* mutations. MRD-negative CR/CRi was achieved in 94% of evaluable patients, and 50% of patients underwent alloHSCT. Median OS has not been reached at a median follow-up of 45 months. Furthermore, translational studies found that no *FLT3* mutant clones were found at relapse in patients who completed protocol therapy.

In considering the role of FLT3i in ND *FLT3*-mutant AML, intensive induction chemotherapy plus MIDO remains a standard of care for eligible patients. However, the formal release of data from QuANTUM-FIRST is awaiting, and ongoing trials will assess other frontline combinations with QUIZ (NCT04209725, NCT04047641), CREN (NCT03258931), and GILT (NCT04027309, NCT03836209), including head-to-head comparisons against MIDO. If QUIZ is approved for ND AML, it should be emphasized that its use would be limited to patients with *FLT3*-ITD, while those with TKD mutations should still receive MIDO. For patients who are ineligible for intensive chemotherapy, AZA/VEN is effective for those with *FLT3-*TKD mutations; unfortunately, better frontline options for those with *FLT3*-ITD are currently limited. Nonetheless, GILT remains a very active FLT3i in the relapsed setting, and early data from doublet and triplet FLT3i combinations are encouraging in ND and R/R AML [143]. Several trials exploring triplet combinations with DAC/VEN/QUIZ (NCT03661307) and AZA/VEN/GILT (NCT04140487) are currently enrolling with results highly anticipated.

## Maintenance therapy and consideration of treatment-free remissions

While the historical focus of induction therapy in AML is to achieve remission and proceed with transplantation in eligible patients, the emergence of tolerable oral therapies posits the role of maintenance therapy, particularly in patients with a higher risk of relapse, such as those with pre-transplantation MRD positivity. There are limited data to support the use of targeted therapies as maintenance after transplant except for SORA. Although SORA has limited efficacy in the frontline setting [144, 145], the SORMAIN trial evaluated SORA maintenance in patients with *FLT3*-ITD after alloHSCT [146]. The investigators noted a 25–30% absolute improvement in 2-year RFS and OS compared to the placebo. Patients treated with SORA had higher rates of GVHD and skin toxicity, consistent with previous reports about its immunogenicity [147]. These results have since been corroborated by another phase 3 study of SORA maintenance post-alloHSCT in CR1 for patients with *FLT3*-ITD [148]; thus, while SORA does not have an FDA label indication for use in the treatment of ND or R/R *FLT3*-mutant AML, it is recommended for use in patients with *FLT3*-ITD who achieve remission after alloHSCT [45]. While no other agents are currently recommended for post-alloHSCT maintenance, the MORPHO trial (NCT02997202) is evaluating the efficacy of GILT versus placebo in this setting for patients with AML and *FLT3* mutations and has completed enrollment.

For patients in remission after induction therapy but unfit for transplant, maintenance options have been limited in the absence of *FLT3*-ITD. The QUAZAR AML-001 study [149] randomized patients who had achieved CR/CRi after intensive chemotherapy but were not fit for alloHSCT to receive oral azacitidine (CC-486) maintenance or placebo. Median OS after randomization was longer in patients receiving CC-486 compared to placebo (24.7 months versus 14.8 months), though it should be noted that only 14% of patients had adverse-risk cytogenetics. Indeed, studies have demonstrated disease-free survival (DFS) but no OS benefit with subcutaneous AZA maintenance in older patients in CR/CRi after induction [150] or with CC-486 in patients with adverse-risk cytogenetics after alloHSCT [151]. Combining VEN with AZA may improve these outcomes based on preliminary data from a phase 2 study (NCT04062266), particularly in those who received VEN-based induction [152]. Furthermore, the VIALE-M study (NCT04102020) is also investigating the role of CC-486 in combination with VEN for patients with CR1 after induction, and the VIALE-T study (NCT04161885) is assessing AZA/VEN maintenance after alloHSCT.

With several options for low-intensity maintenance therapies, the question remains as to whether treatment needs to be indefinite. A retrospective study reported their experience with discontinuing HMA/VEN or LDAC/VEN in transplant-ineligible patients older than 65 years who achieved CRc after receiving either combination for at least 12 months in the frontline setting [153]. Patients who stopped therapy experienced a median treatment-free remission (TFR) of 45.8 months with over half still in remission at end of data collection. No significant differences in RFS or OS were noted between cohorts. One caveat is that patients were highly selected; the vast majority of patients achieved true CR with MRD negativity at the time of discontinuation. Moreover, of patients who sustained treatment-free remission, 86% had a prior *NPM1* or *IDH2* mutation with MRD negativity at cessation. Consequently, in this specific population, MRD negativity may be a reasonable impetus to interrupt treatment, though prospective studies formally evaluating this question are warranted.

## Investigational agents and future directions

**Fig. 4:**
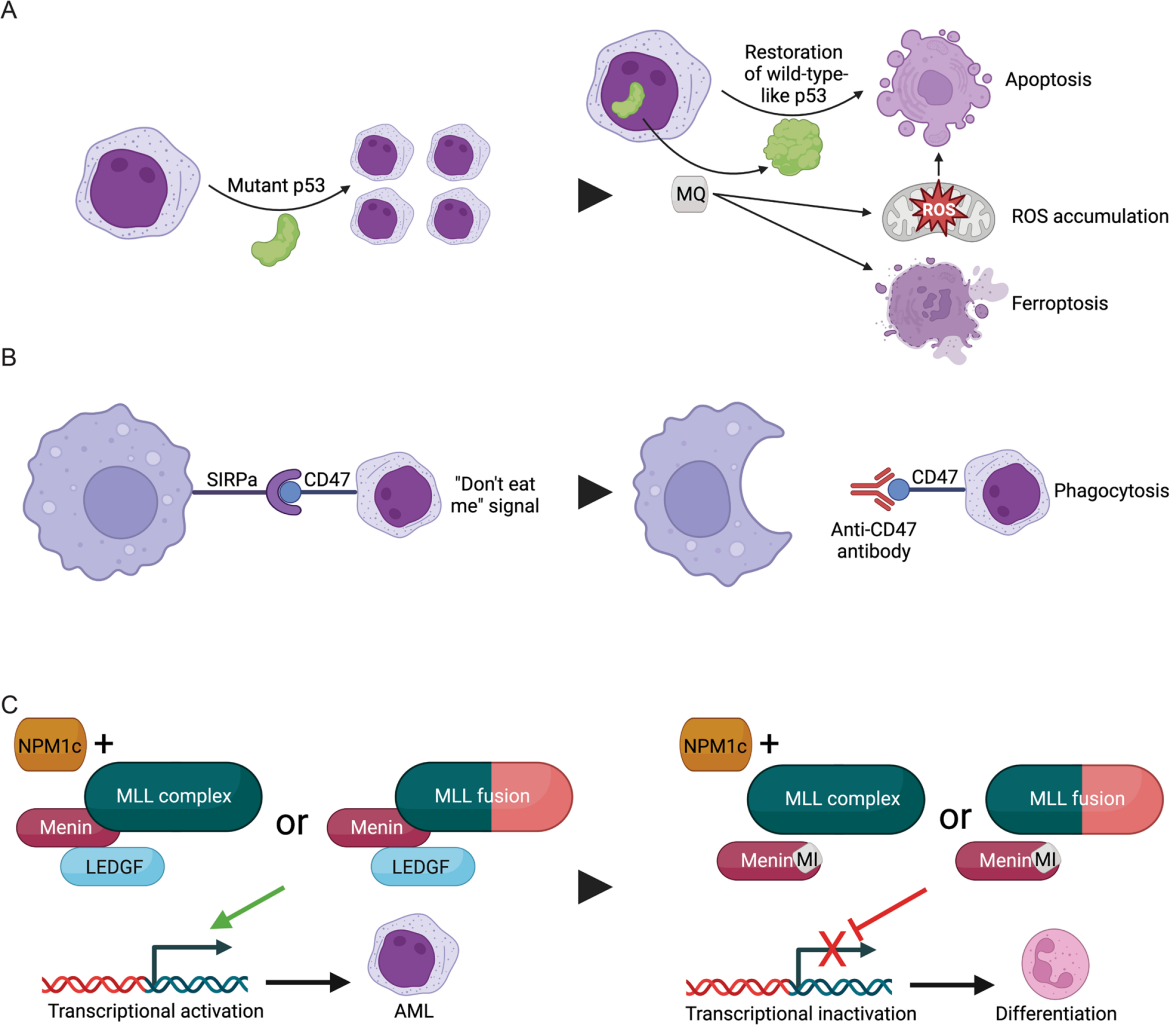
Mechanisms of novel targeted therapies in AML in later-stage clinical development. **A** Mutations in *TP53* lead to altered conformation of p53 leading to a subtype of AML characterized by treatment resistance, high relapse rates, and poor overall survival. Novel agents such as eprenetapopt are metabolized into MQ which covalently modifies the mutant p53 protein leading to a wild-type-like conformational change and restoration of normal p53 activity [155]. Recent reports have demonstrated that MQ can drive p53-independent cell death through ROS accumulation [156, 157] and ferroptosis [158]. **B** Leukemic cells can evade immune surveillance by upregulation of CD47, which binds SIRPa; this emits a “don’t eat me signal” to macrophages [163–165]. Antibodies targeting CD47 can block this inhibitory signal and allow for phagocytosis of leukemic cells [165–167]. **C** Certain types of AML are characterized by mutated NPM1c or oncogenic fusion partners associated with MLL [173, 174, 179]. These lead to complex formation with menin and LEDGF, ultimately resulting in transcriptional activation of leukemia stem cell promoting genes [175–177]. Blocking this pathway with menin inhibitors such as KO-539 or SNDX-5613 can repress this transcriptional program allowing for differentiation of granulocytes [178, 180, 181]. *AML* Acute myeloid leukemia, *MQ* Methylene quinuclidinone, *ROS* Reactive oxidative species, *SIRPa* Signal regulatory protein alpha, *NPM1c* Cytoplasmic NPM1 (mutant NPM1), *MLL* Histone lysine *N-*methyltransferase 2A (KMT2A), *LEDGF* Lens epithelium-derived growth factor, *MI* Menin inhibitor

The aforementioned studies emphasize an increasingly nuanced approach to treatment decision-making in AML as molecular data such as types of mutations (i.e., *FLT3*-ITD versus *FLT3*-TKD) and co-mutational patterns (i.e., *IDH1/2* with *DNMT3A* or RTK pathway mutations) have important prognostic and treatment implications. Although *TP53*-mutant myeloid neoplasms remain one of the largest unmet needs in care, it is encouraging that detailed mechanistic studies may open the door to the development of further targeted therapies. Moreover, with the adoption of genomic data into routine care for patients with AML [18, 154], larger population-based studies will hopefully improve the personalization of treatments. Below we briefly highlight investigational agents (Fig. 4, Table 3), with a focus on those in later clinical development.

**Table 3:** Investigational agents in clinical development for AML and/or MDS

Class/Pathway	Investigational agent	Alternative names	Proposed MOA/Target	AML/MDS trials
FLT3 inhibitor	Quizartinib		Type I FLT3 inhibitor	NCT04107727, NCT03135054, NCT04493138, NCT04128748, NCT03735875, NCT02668653, NCT01892371, NCT03661307, NCT03793478, NCT04047641, NCT04687761, NCT04112589, NCT02984995, NCT03723681, NCT02039726, NCT02834390, NCT01565668, NCT02675478, NCT00462761, NCT01468467, NCT01390337, NCT00989261, NCT01411267
	Crenolanib		Type I FLT3 inhibitor	NCT03258931, NCT02400255, NCT03250338, NCT01657682, NCT01522469, NCT02400281, NCT02626338, NCT02283177, NCT02270788
	Ponatinib		Type I FLT3 inhibitor	NCT02428543, NCT03690115
	Luxeptinib	CG-806	Type I FLT3 inhibitor	NCT04477291
	HM43239		Type I FLT3 inhibitor	NCT03850574
	FF-10101		Type I FLT3 inhibitor	NCT03194685, NCT02193958
IDH inhibitor	LY3410738		IDH1/2 inhibitor	NCT04603001
BCL2/MCL1	S65487	VOB560	BCL2 inhibitor	NCT04742101, NCT03755154, NCT04702425
	S55746	BCL201	BCL2 inhibitor	NCT02920541
	AMG 176		MCL1 inhibitor	NCT05209152, NCT02675452
	AZD5991		MCL1 inhibitor	NCT03013998
	S64315	MIK665	MCL1 inhibitor	NCT04629443, NCT03672695, NCT02979366, NCT02992483, NCT04702425
Mutant P53	Eprenetapopt	APR-246	Mutant p53 reactivation, cellular redox modification, ferroptosis	NCT03931291, NCT04214860, NCT03745716, NCT03072043,
Epigenetic pathways	Ziftomenib	KO-539	Menin-MLL inhibitor	NCT04067336
	Revumenib	SNDX-5613, VTP50469	Menin inhibitor	NCT05406817, NCT05326516, NCT04065399, NCT05360160, NCT03013998
	Pinometostat	EPZ5676	DOT1L inhibitor	NCT03701295
	Iadademstat	ORY-1001	KDM1A inhibitor	NCT05546580
	Entinostat	SYNDX-275, MS-275	HDAC1/3 inhibitor	NCT00101179, NCT00313586, NCT00462605, NCT00015925, NCT02936752
Other oncogenic pathways	Pevonedistat	TAK-924, MLN4924	NEDD8 activator	NCT03459859, NCT03772925, NCT04266795, NCT03813147, NCT03268954
	Entospletinib	GS-9973	Syk inhibitor	NCT05020665, NCT03013998
	Olaparib		PARP inhibitor	NCT03953898
	Milademetan	DS-3032, RAIN-32	MDM2/HDM2 inhibitor	NCT03671564, NCT03634228
	Siremadlin	HDM201	MDM2/HDM2 inhibitor	NCT05447663, NCT05155709
	Uproleselan	GMI-1271	E-selectin antagonist	NCT04964505, NCT05569512, NCT05054543, NCT04839341, NCT04848974, NCT03616470, NCT02306291
	Tamibarotene	SY-1425	Retinoic acid receptor alpha agonist	NCT04797780, NCT04905407, NCT02807558
	JNJ-74856665		Dihydroorotate dehydrogenase inhibitor	NCT04609826
	PRT543		PRMT5 inhibitor	NCT03886831
	H3B-8800	RVT-2001	Splicing modulator	NCT0281540
	Emavusertib	CA-4948	IRAK4 inhibitor	NCT04278768, NCT05178342
Targeted Immune inhibitors	Magrolimab	Hu5F9-G4, ONO-7913	Anti-CD47	NCT04313881, NCT05367401, NCT05079230, NCT04435691, NCT03248479, NCT04778397, NCT04778410, NCT02678338
	Lemzoparlimab	TJ011133, TJC4	Anti-CD47	NCT04202003, NCT04912063
	Ipilimumab		Anti-CTLA4	NCT01757639, NCT02890329, NCT03600155, NCT02397720, NCT03912064, NCT02846376, NCT00060372, NCT01822509, NCT02530463
	Nivolumab		Anti-PD-1	NCT03417154, NCT02530463, NCT02464657, NCT03600155, NCT03358719, NCT03092674, NCT02846376, NCT02532231, NCT02397720, NCT01822509, NCT04913922, NCT03825367, NCT02275533, NCT04277442
	Pembrolizumab		Anti-PD-1	NCT02996474, NCT02845297, NCT02708641, NCT03769532, NCT02768792, NCT02771197, NCT04284787, NCT04214249, NCT03969446, NCT04372706, NCT03761914, NCT03144245, NCT02981914, NCT03094637, NCT02936752, NCT01953692
	Spartalizumab	PDR001	Anti-PD-1	NCT03066648
	Relatlimab		Anti-LAG3	NCT04913922
	Sabatolimab	MBG453	Anti-TIM3	NCT04812548, NCT04623216, NCT04878432, NCT05367401, NCT05201066, NCT04150029, NCT03946670, NCT04266301
Decoy receptor	Evorpacept	ALX148	Anti-CD47	NCT04755244, NCT04417517
ADC	IMGN632		Anti-CD123	NCT03386513, NCT04086264, NCT05320380
	Cusatuzumab	ARGX-110	Anti-CD70	NCT04023526, NCT04241549, NCT04150887, NCT03030612
	Vadastuximab	SGN-CD33A	Anti-CD33	NCT02326584, NCT01902329
BITE/DART	AMV564		CD33 x CD3	NCT03144245, NCT03516591
	AMG 427		FLT3 x CD3	NCT03541369
	Flotetuzumab	MGD006	CD123 x CD3	NCT05506956, NCT04158739, NCT04582864, NCT04681105
	Vibecotamab	XmAB14045	CD123 x CD3	NCT02730312, NCT05285813
	APVO436		CD123 x CD3	NCT03647800

A summary of agents without FDA approval or NCCN recommendation for the treatment of AML/MDS but under investigation for the treatment of AML and/or MDS is shown along with proposed mechanisms of action and clinical trials that are not yet recruiting, recruiting, enrolling, active but not recruiting, or completed. Terminated or suspended studies have been omitted. Cellular therapies (i.e., CAR T cells) are not shown. *MOA* Mechanism of action, *AML* Acute myeloid leukemia, *MDS* Myelodysplastic syndrome, *ADC* Antibody–drug conjugate, *BITE* Bispecific T-cell engager, *DART* Dual affinity retargeting protein

### P53 reactivation

Eprenetapopt (APR-246) is a small-molecular inhibitor originally thought to work through covalent modification of mutated p53, which restores wild-type-like p53 conformation, thus functionally reactivating it [155]. However, its mechanism may also target other synergistic pathways which can drive p53-independent cell death including modulation of cellular redox [156, 157] and increasing glutathione turnover, leading to ferroptosis [158]. Two phase 2 studies are evaluating the use of this agent in combination with AZA in *TP53*-mutant MDS and AML [159, 160]. In these studies, CR rates of patients with MDS were 47–50% with durable responses. In patients with AML, however, CR was only 17%. Notably, responding patients in both studies had significant reductions in *TP53* VAF. Median OS was 10.8–12.1 months for MDS patients and 13.9 months for AML patients. Notable toxicities were febrile neutropenia and neurologic toxicity. Recent data from a phase 2 study (NCT03931291) was presented, which assessed eprenetapopt/AZA as post-alloHSCT maintenance in patients with *TP53*-mutant MDS and AML [161]. Patients received a median of 7 cycles of treatment with a median RFS of 12.5 months and a median OS of 20.6 months. No 30-day mortalities were noted from the first dose. These results, while modest, are encouraging in this very high-risk population. Preclinical studies suggest that XPO1 upregulation may contribute to eprenetapopt resistance in AML and can be overcome with agents like selinexor, though this will require in vivo validation [162]. An additional trial combining eprenetapopt with AZA/VEN has completed enrollment and awaiting data release (NCT04214860). A phase 1 study of APR-548, which is a next-generation molecule, in combination with AZA (NCT04638309) had opened but was terminated by the sponsor.

### CD47—targeting the “don’t eat me” signal

CD47 is a heavily glycosylated cell surface protein and is expressed by virtually all cells in the body, including those that do not express integrin, such as red blood cells [163]. It provides an anti-phagocytic signal in healthy cells but was discovered as an adverse prognostic factor in AML as it is overexpressed on leukemic stem cells compared to non-leukemic stem cells; preclinical murine models demonstrated that blockade of CD47 with monoclonal antibodies could enable phagocytosis of leukemic stem cells and prevent in vivo engraftment [164, 165]. These findings led to the development of a humanized anti-CD47 antibody known as Hu5F9-G4 or magrolimab [166]. A phase 1b study explored the tolerability of magrolimab in combination with AZA (NCT03248479) for patients with untreated intermediate- to very high-risk MDS and with untreated AML unfit for intensive chemotherapy [167]. *TP53* mutations were noted in 27% of patients. Common adverse events included anemia, neutropenia, thrombocytopenia, and infusion reactions. In transfusion-dependent MDS and AML patients, 58% and 64% were able to achieve transfusion independence; moreover, CR/CRi rate was 56% in AML patients. The median duration of response was not reached in MDS, AML, or *TP53-*mutant AML subpopulations. Preliminary data from a phase 1b/2 study evaluating the combination of magrolimab/AZA/VEN (NCT04435691) noted CR/CRi rates of 63% and 86% in patients with ND AML with or without *TP53* mutations, respectively, conferring 1-year OS rates of 53% and 83%, respectively [168]. In patients with R/R AML, median OS was only 7.4 months with responses especially limited in those with prior VEN exposure. Given these findings, particularly in the very high-risk *TP53*-mutant group, three phase 3 trials opened which are comparing magrolimab/AZA versus AZA in patients with untreated intermediate- to very high-risk MDS (NCT04313881), magrolimab/AZA versus AZA/VEN or intensive chemotherapy in patients with untreated *TP53*-mutant AML (NCT04778397), and magrolimab/AZA/VEN versus AZA/VEN in patients with untreated AML who are ineligible for standard intensive chemotherapy (NCT05079230). A phase 2 study of magrolimab with various anti-leukemic therapies in patients with untreated AML is enrolling as well (NCT04778410). An additional combination study of another anti-CD47 antibody, lemzoparlimab, had opened for patients with a higher-risk MDS or AML ineligible for intensive chemotherapy (NCT04912063) but was recently stopped.

### Menin inhibition

Several types of AML have overexpression of *HOXA/B* cluster genes and *MEIS1*, which are critical regulators of hematopoietic stem cell self-renewal and differentiation [169–171]. These can be dysregulated with alterations of histone lysine *N-*methyltransferase 2A (KMT2A or MLL) and/or *NPM1* mutations [172–174]. KMT2A binds menin as part of a histone methyltransferase complex, and when it is involved in an oncogenic fusion, it may lead to aberrant transactivation of leukemia-promoting genes [175–177]. While mechanistically unclear, cytoplasmic localization of the mutant NPM1 is associated with a similar phenotype and genetic signature as KMT2A-driven AML [173, 178, 179]. Consequently, several preclinical models examined the role of menin inhibition in these subtypes of AML and observed efficacy [178, 180, 181]. This has led to the opening of multiple phase 1/2 studies in patients with R/R AML with KMT2A-rearrangement or *NPM1*-mutation using the menin-MLL inhibitor KO-539 (NCT04067336) or menin inhibitor SNDX-5613 (NCT04065399, NCT05326516, NCT05406817, NCT05360160, NCT03013998) which are currently enrolling patients or opening soon. Early data from NCT04067336 and NCT04065399 suggest similar CR/CRh rates (25–30%) and MRD negativity rates in responding patients (75–78%) with either KO-539 or SNDX-5613, respectively [182–184]. Toxicities include QTc prolongation with SNDX-5613 and differentiation syndrome, particularly with KO-539. Of note, however, the activity of KO-539 appears largely restricted to patients with *NPM1*-mutated AML, in whom differentiation syndrome was not observed.

### Other therapies in clinical development

In addition to the aforementioned therapies, several other pathways have been identified as possible therapeutic vulnerabilities in AML and are emerging in early clinical development [185, 186]. Some of these agents target epigenetic and oncogenic signaling pathways and may allow for the augmentation of available therapies. The ALICE study is evaluating the use of iadademstat, a lysine-specific demethylase 1 inhibitor, in combination with AZA for the frontline treatment of AML in patients unfit for intensive chemotherapy. Preliminary data suggest CR/CRi rates of 64% with median OS extending to 14.3 months in responding patients. Strikingly, 75% of patients with *TP53* alterations responded [187]. There has also been a considerable interest in exploiting advances in immunotherapy and cellular therapy for the treatment of AML. Unfortunately, findings from early studies on these agents have been underwhelming thus far. Flotetuzumab is a dual affinity retargeting antibody that targets CD123 and CD3. A phase 1/2 study reported flotetuzumab could achieve CR/CRh/CRi rates of 30% with 12-month OS of 75% in patients with R/R AML [188] with recent data suggesting similar outcomes in pediatric and adolescent/young adult patients [189]. The majority of patients who benefit from flotetuzumab are primary refractory and *TP53-*mutated, which is related to a distinct immune microenvironment compared to non-*TP53-*mutated AML. An ongoing phase 1b/2 study is also evaluating the combination of pivekimab sunirine (IMGN632), a CD123-targeting antibody–drug conjugate, with AZA/VEN [190]. Patients with R/R AML treated with this were reported to have CRc rates of 31%, including 26% in those with ELN adverse disease and 64% of those with *FLT3-*ITD, with minimal additive myelosuppression beyond that of AZA/VEN. In regard to checkpoint inhibition, findings from the unpublished REMAIN trial did not demonstrate a PFS or OS benefit with the use of nivolumab maintenance in patients with CR/CRi who are ineligible for alloHSCT [191]. Nevertheless, immune dysregulation is important in AML, though it is not yet clear how to effectively target the tumor microenvironment or which factors can be used to predict response to immune-based therapies.

## Conclusion

AML is a very complex and heterogeneous disease as evidenced by the expansion of genetic and cytogenetic qualifiers in the updated WHO [23] and ICC [24] classification systems. While outcomes for AML continually improve by decade, a refined understanding of patient and tumor characteristics is needed to continue this upward trend. This is especially true when selecting patients for intensive chemotherapy and/or alloHSCT, as a standardized and validated metric of physiologic age or fitness would greatly improve our ability to personalize treatments and design clinical trials more representative of the actual patient population. However, we also now have several active, low-intensity therapies approved or in the developmental pipeline. Perhaps, the most transformative has been the combination of AZA/VEN, which has supplanted conventional cytotoxic chemotherapy in many cases. Unfortunately, outcomes in patients who progress on HMA/VEN are poor, particularly in patients who harbor *TP53* or RAS-pathway mutations [192], and clinical development of additional therapeutic options is critical. Moreover, several groups will soon be reporting data from trials investigating the addition of VEN to induction chemotherapy in both the ND and R/R AML setting to answer the question if there is any benefit to further intensification of therapy [193–196].

We are entering a unique era of precision oncology whereby molecularly informed data can be exploited to tailor treatments based on disease pathobiology. But while options have rapidly increased for patients *IDH1/*2 and *FLT3* mutations, progress has unfortunately been slow for those with the highest risk forms of AML, such as *TP53*-mutant disease. Nevertheless, the incorporation of data such as co-mutational burden and MRD analysis will hopefully allow us to better define patients at the highest risk of relapse [197–199] and who may benefit from early relapse intervention [200–202]. Pertinent questions in this regard will be whether induction of deeper molecular remissions with combination therapies would improve outcomes compared to sequencing therapies, how we can minimize toxicity associated with combinations of novel agents, and if we can use MRD negativity to interrupt treatment.

The multitude of active studies addressing these questions will lend nuance to clinical practice and contribute to improved outcomes for patients.

## Data Availability

The material supporting the information of this review has been included within this article.

## Abbreviations

2-HG: 2-Hydroxyglutarate
AlloHSCT: Allogeneic stem cell transplantation
AML: Acute myeloid leukemia
AR: Allelic ratio
AZA: Azacitidine
bZIP: Basic leucine zipper
CR: Complete remission
CR1: First remission
CRc: Composite complete remission
CREN: Crenolanib
CRh: Complete response with partial hematologic recovery
CRi: Complete remission with incomplete hematologic recovery
CRp: Complete remission with incomplete platelet recovery
DAC: Decitabine
DOR: Duration of remission
dPCR: Digital polymerase chain reaction
EB2: Excess blasts-2
EFS: Event-free survival
ELN: European LeukemiaNet
ENA: Enasidenib
FDA: Food and drug administration
FLT3i: FLT3 inhibitor
GILT: Gilteritinib
HCT-CI: Hematopoietic cell transplantation-specific comorbidity index
HiDAC: High-dose cytarabine
HMA: Hypomethylating agent
ICC: International consensus classification
ITD: Internal tandem duplication
IVO: Ivosidenib
KMT2A/MLL: Histone lysine N-methyltransferase 2A
LDAC: Low-dose cytarabine
MDS: Myelodysplastic syndrome
MIDO: Midostaurin
MRD: Measurable residual disease
NCCN: National comprehensive cancer network
ND: Newly diagnosed
NGS: Next-generation sequencing
OLU: Olutasidenib
OS: Overall survival
QUIZ: Quizartinib
R/R: Relapsed/refractory
RFS: Recurrence-free survival
RTK: Receptor tyrosine kinase
sAML: Secondary acute myeloid leukemia
SORA: Sorafenib
tAML: Therapy-related AML
TFR: Treatment-free remission
TKD: Tyrosine kinase domain
TKI: Tyrosine kinase inhibitor
VAF: Variant allelic frequency
VEN: Venetoclax
WHO: World health organization
